# Identification and Validation of Potential Candidate Genes of Colorectal Cancer in Response to *Fusobacterium nucleatum* Infection

**DOI:** 10.3389/fgene.2021.690990

**Published:** 2021-09-28

**Authors:** Jiangguo Zhang, Zhimo Wang, Hong Lv, Guojun Li

**Affiliations:** ^1^ Department of Gastroenterology, Shenzhen Shekou People’s Hospital, Shenzhen, China; ^2^ Department of Gastroenterology, Shenzhen Nanshan People’s Hospital, Shenzhen, China; ^3^ Department of Liver Disease, Shenzhen Third People’s Hospital, Shenzhen, China

**Keywords:** *Fusobacterium nucleatum*, colorectal cancer, cell cycle, apoptosis, bioinformatics analysis, validation

## Abstract

**Objective:** Recent investigations revealed the relationship between *Fusobacterium nucleatum* (Fn) infection and colorectal cancer (CRC). However, how the host genes changes contribute to CRC in response to Fn infection remains largely unknown.

**Materials and methods:** In the present study, we aimed to comprehensively analyze microarray data obtained from a Caco-2 infection cell model using integrated bioinformatics analysis and further identify and validate potential candidate genes in Fn-infected Caco-2 cells and CRC specimens.

**Results:** We identified 10 hub genes potentially involved in Fn induced tumor initiation and progression. Furthermore, we demonstrated that the expression of centrosomal protein of 55 kDa (CEP55) is significantly higher in Fn-infected Caco-2 cells. Knocking down of CEP55 could arrest the cell cycle progression and induce apoptosis in Fn-infected Caco-2 cells. The expression of CEP55 was positively correlated with the Fn amount in Fn-infected CRC patients, and these patients with high CEP55expression had an obviously poorer differentiation, worse metastasis and decreased cumulative survival rate.

**Conclusion:** CEP55 plays an important role in Fn-infected colon cancer cell growth and cell cycle progression and could be used as a new diagnostic and prognostic biomarker for Fn-infected CRC.

## Introduction

Many malignant cancers are characterized by complex communities of oncogenic potentially transformed cells with genetic and epigenetic changes caused by bacteria and viruses (Burnett-Hartman et al., 2008). *Fusobacterium nucleatum* (Fn) is a gram-negative obligate anaerobic bacterium that could adhere to and invade endothelial or epithelial cells through its adhesin FadA. The aggregation of Fn in intestinal epithelium promotes the occurrence and development of colorectal adenoma and adenocarcinoma (Flanagan et al., 2014; Park et al., 2016; Yan et al., 2017; Yamaoka et al., 2018). It has been found that FadA can binds to vascular endothelial adhesion factor CDH5 and activate p38MAPK signal pathway to promote the progress of colorectal cancer (CRC) (Rubinstein et al., 2013). FadA can also bind with E-cadherin on epithelial cells and activate oncogenes Myc and Cyclin D1. Recent studies indicated that Fn can bind to TLR4 with its lipopolysaccharide and activate the cascade reaction of p-pak-1/P-β-Catenins-675/c-myc/Cyclin-D1 to promote the malignant proliferation of colon cancer cells (Chen et al., 2017; Yang et al., 2017). Furthermore, it was found that Fn could enhance the growth and migration of CRC cells by the overexpression of microRNA-21 through TLR4/NF-κB signaling pathway (Yang et al., 2017). Although these factors are associated with the carcinogenesis induced by Fn, still little is known about genes that contribute to CRC in Fn infection microenvironment.

Recently, the high-throughput gene microarray analysis of Fn-infected and non-infected Caco-2 cells allows us to explore the global molecular changes from transcriptome alterations to somatic mutations, as well as epigenetic changes (De et al., 2015; Jia et al., 2017). In this study, the GSE102573 dataset from the Gene Expression Omnibus (GEO, http://www.ncbi.nlm.nih.gov/geo) database was downloaded and the differentially expressed genes (DEGs) were comprehensively identified using GEO2R. Then, a protein-protein interaction (PPI) network of these DEGs was established and 10 hub genes with a high degree of connectivity were screen out. In addition, Gene Ontology (GO) involving the biological processes (BPs), molecular functions (MFs), and cellular components (CCs) of these DEGs and their Kyoto Encyclopedia of Genes and Genomes (KEGG) pathways were also analyzed. The potential correlation and expression levels were further analyzed via Gene Expression Profiling Interactive Analysis (GEPIA) (http://gepia.cancer-pku.cn/index.html) and validated via quantitative reverse transcription-PCR (qRT-PCR).

Our data showed that the expression of centrosomal protein of 55 kDa (CEP55) is significantly higher in Fn-infected Caco-2 cells. Knocking down of CEP55 could arrest the cell cycle progression and induce apoptosis in Fn-infected Caco-2 cells. The expression of CEP55 was positively correlated with the Fn amount in Fn-infected CRC patients, and these patients with high CEP55 expression had an obviously poorer differentiation, worse metastasis and decreased cumulative survival rate.

## Materials and Methods

### Microarray Data

The gene expression profile of GSE102573 was downloaded from the GEO free public database. This microarray dataset has a total of 5 Fn-infected and 5 Fn-non-infected Caco-2 samples and was based on the Agilent GPL17586 platform [Affymetrix Human Transcriptome Array 2.0 (transcript (gene) version)].

### Data Preprocessing

All of the probes expression values in each sample were reduced to a single mean expression value via the aggregate function method and missing data were assigned using the k-nearest neighbor method (Li, 1991; Altman, 1992). When many genes were located by a probe, the probe was considered to be lack of specificity and was removed from the analysis.

### Identification of DEGs

GEO2R was utilized to identify the DEGs between Fn-infected and Fn-non-infected Caco-2 samples. The adjusted *p*-value, which could help correct false positives, was applied and adjusted *p* < 0.01 and |log fold change (FC)| > 1 were chosen as the cutoff criteria. The heat map and volcano plot were drawn using the “gplots” package in R 3.5.3 (Ge et al., 2021; Ritchie et al., 2015). A total of 272 upregulated genes and 178 downregulated genes were found and the top 10 genes with a high degree of connectivity were selected as hub genes.

### GO and KEGG Pathway Analysis of DEGs

GO analysis can be used to annotate genes and their products with CCs, MFs, BPs, and other functions (Gaudet et al., 2017; Ning et al., 2013). The KEGG databases address genomic and biological pathways related to diseases and drugs and provide a comprehensive understanding of biological systems and genomic functional information (Kanehisa, 2002). DAVID (http://david.ncifcrf.gov) (version 6.8) can integrate large amounts of biological data and related analysis tools to provide systematic and comprehensive biological function annotation information for high-throughput gene expression (Huang et al., 2007).To visualize the key CCs, MFs, BPs and KEGG pathways of the DEGs, the DAVID online database was used to perform biological analysis. *p* < 0.05 was used as the cut-off criterion for statistically significant differences.

### PPI Network and Module Analysis

The Search Tool for the Retrieval of Interacting Genes/Proteins (STRING) version 11.0 is used to evaluate and integrate physical and functional PPI information (Szklarczy et al., 2015). The network of DEGs in STRING was drawn to evaluate the interrelationships among these DEGs and the PPI network was visualized by using Cytoscape software. Moreover, the maximum number of interacting bodies to 0 and a confidence score of 0.7 as the cut-off criterion were used. Additionally, according to node score cut-off = 0–2, degree cut-off = 2, max. depth = 100, and k-core = 2, the Molecular Complex Detection (MCODE) app was also employed to select the PPI network modules in Cytoscape and the top three modules were analyzed with DAVID.

### The Expression and Survival Analysis of the 10 Hub Genes

GEPIA is a newly developed interactive web server designed to accurately analyze the RNA sequence expression data of 9,736 tumors and 8,587 normal samples from the TCGA and GTEx projects (Tang et al., 2017). The top 10 hub genes’ correlation was analyzed using GEPIA tool. Then, boxplots were used to visualize hub gene expression in CRC and normal colon tissues in our study. The disease-free survival analysis of the 10 hub genes was also obtained from GEPIA. Gene Set Cancer Analysis (GSCA) is a website that collects the cancer genomics data of 33 cancer types from TCGA database (Liu C.-J. et al., 2018; Liu J. et al., 2018). The top 10 hub genes’ expression in different CRC stages was analyzed using GSCA tool.

### Validation Based on Fn-Infected and Fn-Non-Infected Caco-2 Cells

To further verify the data from GEO, qRT-PCR was conducted to quantify the expression level of 10 hub genes in Fn-infected and Fn-non-infected Caco-2 cells. We adhered to standard biosecurity and institutional safety procedures of Shenzhen Qianhai and Shekou Free Trade Zone’s hospital. Caco-2 cells (Bioyear Biotechnology, China) were cultured in RPMI-1640 medium containing 100 U/ml streptomycin/penicillin and 10% FBS (Thermo Fisher Scientific, United States). Fn (ATCC 25586) was used in the culture of Fn-infected Caco-2 cells as described before (Jia et al., 2017). All the Fn-infected and Fn-non-infected Caco-2 cells were maintained in a humidified incubator with 5% CO_2_ at 37°C. Total RNA was extracted from cells using TRIzol reagent (Invitrogen, United States). Reverse-transcribed complementary DNA was synthesized with the Prime Script RT Reagent Kit (Takara, Japan) and the RT-PCR conditions were as follows: 37°C for 15 min, 85°C for 5 s, and held at 4°C. The qRT-PCR was performed by a StepOne™ Real-Time PCR system and SYBR^®^ Premix Ex Taq™, and the qRT-PCR conditions were setup as follows: 1 min at 95°C, followed by 40 cycles at 95°C for 15 s, and 72°C for 45 s. The results were normalized to GAPDH expression. The relative expression level of 10 hub genes was calculated by the 2-ΔCt method. The primers used for qRT-PCR were as follows: GAPDH forward, 5′-CAT​CAT​CCC​TGC​CTC​TAC​TGG-3′, and reverse, 5′-GTG​GGT​GTC​GCT​GTT​GAA​GTC-3′; MAD2L1 forward, 5′-GCA​AAA​GAT​GAC​AGT​GCA​CCC-3′, and reverse, 5′-GTG​GTC​CCG​ACT​CTT​CCC​AT-3′; ANLN forward, 5′-TCC​AAA​GAA​GAT​AAA​AAG​GGG-3′, and reverse,5′-CTGTGCGAACCAGCAACTT-3′; CCNB1 forward, 5′-GAC​CTG​TGT​CAG​GCT​TTC​TCT​G-3′, and reverse, 5′-GGT​ATT​TTG​GTC​TGA​CTG​CTT​GC-3′; CDK1 forward, 5′-CAG​AGA​TTG​ACC​AGC​TCT​T-3′, and reverse, 5′-GAA​AGG​TGT​TCT​TGT​AGT​CC-3′; CEP55 forward, 5′-TTG​GAA​CAA​CAG​ATG​CAG​GC-3′, and reverse, 5′-GAG​TGC​AGC​AGT​GGG​ACT​TT-3′; MELK forward, 5′-GCT​GCA​AGG​TAT​AAT​TGA​TGG​A-3′, and reverse, 5′-CAG​TAA​CAT​AAT​GAC​AGA​TGG​GC-3′; PRC1 forward, 5′-TAG​ACC​ACA​CCC​CAG​ACA​CA-3′, and reverse, 5′-GTG​GCC​ACA​GCT​TCT​CTT​TC-3′; KIF4A forward, 5′-CTG​CAA​TTG​GTT​GGC​GTC​TC-3′, and reverse, 5′-CAG​CGC​CAC​TCT​TAC​AGG​AA-3′; TPX2 forward, 5′-TCC​TGC​CCG​AGT​GAC​TAA​GG-3′, and reverse, 5′-CTG​TTA​GGG​GTT​CGT​TTA​TGG​AA-3′; TRIP13 forward, 5′-CTG​TCT​CTG​GCA​GTG​GAC​AAG-3′, and reverse, 5′-TTG​GTT​TGC​AGA​AGG​GAT​TC-3′.

### Gene Set Enrichment Analysis

Gene Set Enrichment Analysis (GSEA) could be used to explore whether a given gene set is significantly enriched in a group of gene markers which is ranked by their relevance with a phenotype of interest. The gene sets with fewer than 15 genes or more than 500 genes were excluded and the phenotype label was set as colon cancer versus control. The t-statistic mean of the genes was computed in each KEGG pathway by a permutation test with 1,000 replications. The upregulated pathways were defined by a normalized enrichment score (NES) > 0, and the downregulated pathways were defined by an NES < 0. Pathways with a false discovery rate (FDR) < 5% (q < 0.05) were considered significantly enriched.

### TIMER Database Analysis

A comprehensive website TIMER was used for the automatic analysis and visualization of association between immune infiltrate levels and a series of variables (https://cistrome.shinyapps.io/timer/) (Li et al., 2017; Jiang et al., 2018; Wang et al., 2021). We assessed the correlation of CEP55 expression with the abundance of six kinds of immune cells (B cells, CD8 + T cells, neutrophils, dendritic cells, CD4 + T cells and macrophages) in CRC via the TIMER algorithm.

### Caco-2 Cells and Cell Transfection

Fn-infected and non-infected Caco-2 cells were seeded at a density of 1 × 10^4^cells/mlRPMI-1640 medium for 24 h, respectively. Once the cells reached 40–60% confluence in each well of a 96-well plate, the cells were transfected with 2.5 nM siRNA/NC (RiboBio, Guangzhou, China) using Lipofectamine 2,000 (Thermo Fisher Scientific, United States) according to the manufacturer’s instructions. The culture medium was replaced with fresh medium containing 10% FBS 6 h later. The cells were then harvested after 24 h of transfection for the following assays.

The siRNA sequences were as follows: Si-h-CEP55 si#1: 5′-GGG​AGA​AAT​TGC​ACA​CTT​Att-3′; si#2: 5′-GGACTTTTAGCAAAGATCTtt-3′(Chen et al., 2019).

### Cell Proliferation and Apoptosis Analysis

24 h after siRNA interference, Fn-infected and non-infected Caco-2 cells were treated as indicated and cell proliferation was assessed by Cell Counting Kit-8 (CCK-8) assay (Beyotime biotechnology, China) at 0, 24 and 48 h post treatment following the manufacturer’s instruction, respectively. Optical density (OD) was recorded at 450 nm.

Fn-infected and non-infected Caco-2 cells were then harvested, centrifuged, and resuspended in 1×PBS, respectively. The cells were fixed in 70% ethanol overnight. On the second day, after being washed with 1×PBS solution and centrifuged, the cells were resuspended in 1×PBS solution and incubated with RNase A at 37°C for 30 min. Finally, the cells were stained with propidium iodide and analyzed by a FACSCalibur system (BD Biosciences, Germany) for cell cycle analysis.

Fn-infected Caco-2 cells were transfected with siRNA for 24 h, harvested, and centrifuged. Then, the supernatant was removed and resuspended in 1×PBS solution. This procedure was repeated three times with 1 × 10^6^ cells per well, and then the cells were stained with an Annexin V/FITC and PI kit. After staining, the cells were also analyzed with a FACSCalibur system (BD Biosciences, Germany) for apoptosis analysis.

Validation the expression of CEP55 in Fn-infected CRC samples QRT-PCR was conducted to quantify the expression level of CEP55 in Fn-infected CRC samples (*n* = 30) from Shenzhen Qianhai and Shekou Free Trade Zone’s hospital (Shekou people’s hospital, Shenzhen, Guangdong, China). This study was approved by the Ethics Committee of Shenzhen Qianhai and Shekou Free Trade Zone’s hospital, and written informed consent was obtained from each patient before inclusion in the study. The primers used for qRT-PCR were described as before. Written informed consents were obtained from all patients. This study was approved by the Ethics Committee of Shenzhen Qianhai and Shekou Free Trade Zone’s hospital (Shekou People’s Hospital, Shenzhen, Guangdong, China). The correlation between CEP55 expression and Fn amount, cumulative survival rate of Fn-infected CRC samples was calculated and analyzed according to the method described by Aalen (Aalen, 1988). The CEP55 expression and clinicopahtologic features of Fn-infected CRC were also further analyzed.

### Statistical Analysis

All experiments were carried out in triplicate for each condition under the protocol and according to the manufacturer’s instructions. Clinical characteristics were compared using the Wilcoxon rank sum test; categorical variables were compared using the Fisher exact test. Pearson’s correlation test was used to analyze the correlation between the CEP55 expression and Fn amount. Log-rank test was used to determine the association of high/low CEP55 expression with clinical characteristics. Cumulative survival rates were summarized using the Kaplan-Meier method. If *p* < 0.05, these differences were considered to be statistically significant.

## Results

### Identification of DEGs and Hub Genes

**FIGURE 1:**
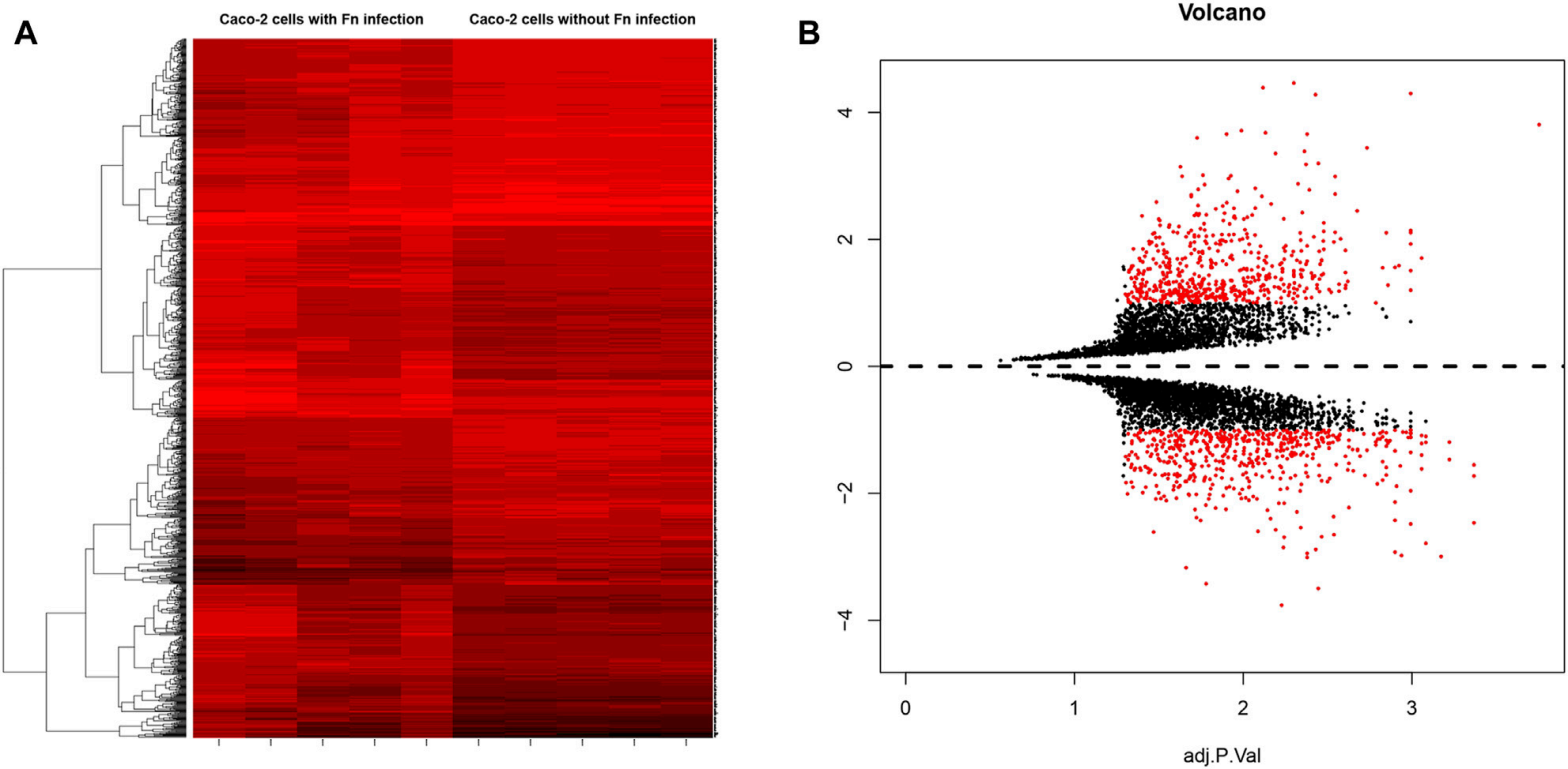
**(A)** Heat map of DEGs. **(B)** Volcano plot of genes detected in Fn-positive and Fn-negative Caco-2 cells. Red means upregulated and downregulated DEGs; black means no difference.

A total of 5 groups of Fn-infected and 5 groups of Fn-non-infected Caco-2 cells were analyzed. The series from each chip was analyzed separately using GEO2R and R software, and the DEGs were identified using adjusted *p* value < 0.01 and logFC ≥ 1 or logFC ≤ −1 as the cut-off criteria. A total of 450 DEGs were identified after analyzing GSE102573, 272 of which were upregulated genes, and 178 were downregulated (Figure 1). In addition, 10 hub genes were identified according to their degree of connectivity, namely CDK1, CCNB1, MAD2L1, CEP55, TPX2, MELK, TRIP13, KIF4A, PRC1 and ANLN (Table 1).

**TABLE 1:** Top 10 hub genes with higher degree of connectivity.

Gene	Degree of connectivity	Adjusted *p* value
CDK1	42	7.19E-28
CCNB1	39	3.65E-21
MAD2L1	35	3.37E-33
CEP55	35	4.54E-31
TPX2	33	7.35E-27
MELK	33	4.24E-28
TRIP13	30	3.01E-28
KIF4A	30	2.19E-26
PRC1	29	2.43E-23
ANLN	29	3.25E-22

### GO Function and KEGG Pathway Enrichment Analysis

To obtain a comprehensive understanding of the selected DEGs, the GO function and KEGG pathway enrichment were analyzed by DAVID. After importing all DEGs into DAVID, we discovered the functions of the upregulated DEGs and downregulated DEGs by GO analysis. More specifically, these DEGs were mainly enriched in BPs involving cell cycle phase, cell cycle process, cell cycle, M phase and mitotic cell cycle for the upregulated genes; and cell adhesion, biological adhesion, ion homeostasis for the downregulated genes. Regarding MFs, the DEGs were involved in nucleoside binding, adenyl nucleotide binding, purine nucleoside binding for the upregulated genes; and polysaccharide binding, pattern binding, calmodulin binding for the downregulated genes. In addition, GO CC analysis revealed that the upregulated DEGs were principally enriched in the organelle lumen, intracellular organelle lumen, membrane-enclosed lumen, endoplasmic reticulum membrane and endoplasmic reticulum, while the downregulated DEGs were mainly enriched in extracellular region part, proteinaceous extracellular matrix, extracellular matrix, extracellular region and calcineurin complex (Table 2).

**TABLE 2:** Gene ontology analysis of differentially expressed genes associated with Fn-infected Caco-2 cells.

Expression	Category	Term	Count	%	*p*Value	FDR
Upregulated	GOTERM_BP_DIRECT	GO:0022403∼cell cycle phase	26	9.85	1.32E-08	2.21E-05
	GOTERM_BP_DIRECT	GO:0000279∼M phase	23	8.71	1.70E-08	2.84E-05
	GOTERM_BP_DIRECT	GO:0022402∼cell cycle process	28	10.61	4.15E-07	6.95E-04
	GOTERM_BP_DIRECT	GO:0007049∼cell cycle	33	12.50	8.73E-07	0.01
	GOTERM_BP_DIRECT	GO:0000278∼mitotic cell cycle	21	7.95	2.30E-06	0.01
	GOTERM_CC_DIRECT	GO:0043233∼organelle lumen	50	18.94	2.32E-05	0.03
	GOTERM_CC_DIRECT	GO:0070013∼intracellular organelle lumen	49	18.56	2.74E-05	0.04
	GOTERM_CC_DIRECT	GO:0031974∼membrane-enclosed lumen	50	18.94	3.90E-05	0.05
	GOTERM_CC_DIRECT	GO:0005789∼endoplasmic reticulum membrane	14	5.30	2.13E-04	0.29
	GOTERM_CC_DIRECT	GO:0005783∼endoplasmic reticulum	30	11.36	2.30E-04	0.31
	GOTERM_MF_DIRECT	GO:0001882∼nucleoside binding	46	17.42	6.59E-05	0.10
	GOTERM_MF_DIRECT	GO:0030554∼adenyl nucleotide binding	45	17.05	8.21E-05	0.12
	GOTERM_MF_DIRECT	GO:0001883∼purine nucleoside binding	45	17.05	1.17E-04	0.17
	GOTERM_MF_DIRECT	GO:0005524∼ATP binding	42	15.91	1.70E-04	0.25
	GOTERM_MF_DIRECT	GO:0032559∼adenyl ribonucleotide binding	42	15.91	2.27E-04	0.33
Downregulated	GOTERM_BP_DIRECT	GO:0007155∼cell adhesion	20	11.70	1.96E-05	0.03
	GOTERM_BP_DIRECT	GO:0022610∼biological adhesion	20	11.70	2.00E-05	0.03
	GOTERM_BP_DIRECT	GO:0050801∼ion homeostasis	15	8.77	2.25E-05	0.04
	GOTERM_BP_DIRECT	GO:0055080∼cation homeostasis	12	7.02	6.34E-05	0.10
	GOTERM_BP_DIRECT	GO:0006875∼cellular metal ion homeostasis	10	5.85	7.91E-05	0.13
	GOTERM_CC_DIRECT	GO:0044421∼extracellular region part	26	15.20	1.41E-05	0.02
	GOTERM_CC_DIRECT	GO:0005578∼proteinaceous extracellular matrix	14	8.19	2.64E-05	0.03
	GOTERM_CC_DIRECT	GO:0031012∼extracellular matrix	14	8.19	5.75E-05	0.07
	GOTERM_CC_DIRECT	GO:0005576∼extracellular region	38	22.22	2.19E-04	0.28
	GOTERM_CC_DIRECT	GO:0005955∼calcineurin complex	3	1.75	0.01	1.31
	GOTERM_MF_DIRECT	GO:0030247∼polysaccharide binding	8	4.68	9.01E-04	1.21
	GOTERM_MF_DIRECT	GO:0001871∼pattern binding	8	4.68	9.01E-04	1.21
	GOTERM_MF_DIRECT	GO:0005516∼calmodulin binding	7	4.09	0.01	3.75
	GOTERM_MF_DIRECT	GO:0030246∼carbohydrate binding	10	5.85	0.01	11.76
	GOTERM_MF_DIRECT	GO:0004364∼glutathione transferase activity	3	1.75	0.02	20.47

Table 3 shows the most significantly enriched KEGG pathways of the upregulated and downregulated DEGs. The upregulated DEGs were enriched in the cell cycle, one carbon pool by folate, while the downregulated DEGs were enriched in axon guidance, chemokine signaling pathway.

**TABLE3:** KEGG pathway analysis of differentially expressed genes associated with Fn-infected Caco-2 cells.

Expression	Term	Count	%	*p* value		FDR
Upregulated	hsa04110:Cell cycle	7	2.65	0.04	CCNB1, CDK1, CDC6, CCND1, MAD2L1, PCNA, CDC25A	35.94
	hsa00670:One carbon pool by folate	3	1.14	0.04	MTHFD2, SHMT2, GART	36.72
	hsa00100:Steroid biosynthesis	6	2.27	1.50E-05	EBP, CYP51A1, SQLE, DHCR7, DHCR24, NSDHL	0.02
	hsa00480:Glutathione metabolism	5	1.89	0.02	GSS, GPX2, GGCT, PGD, IDH1	17.89
	hsa00051:Fructose and mannose metabolism	4	1.52	0.03	SORD, GMDS, TSTA3, PMM2	28.96
Downregulated	hsa04360:Axon guidance	6	3.51	0.01	SEMA6D, SEMA3D, PPP3CB, CXCL12, SLIT3, EPHA3	14.64
	hsa04062:Chemokine signaling pathway	7	4.09	0.02	CCL11, CCL2, RAP1A, GNG2, CXCL12, AKT3, PRKCB	17.72
	hsa00480:Glutathione metabolism	4	2.34	0.02	GSTM1, GSTM2, GPX3, GSTM5	18.26
	hsa00980:Metabolism of xenobiotics by cytochrome P450	4	2.34	0.03	GSTM1, GSTM2, ADH1B, GSTM5	27.92
	hsa00982:Drug metabolism	4	2.34	0.03	GSTM1, GSTM2, ADH1B, GSTM5	30.01
	hsa04720:Long-term potentiation	4	2.34	0.04	PPP3CB, RAP1A, ITPR1, PRKCB	36.43

### Hub Genes and Module Screening of the PPI Network

**FIGURE 2:**
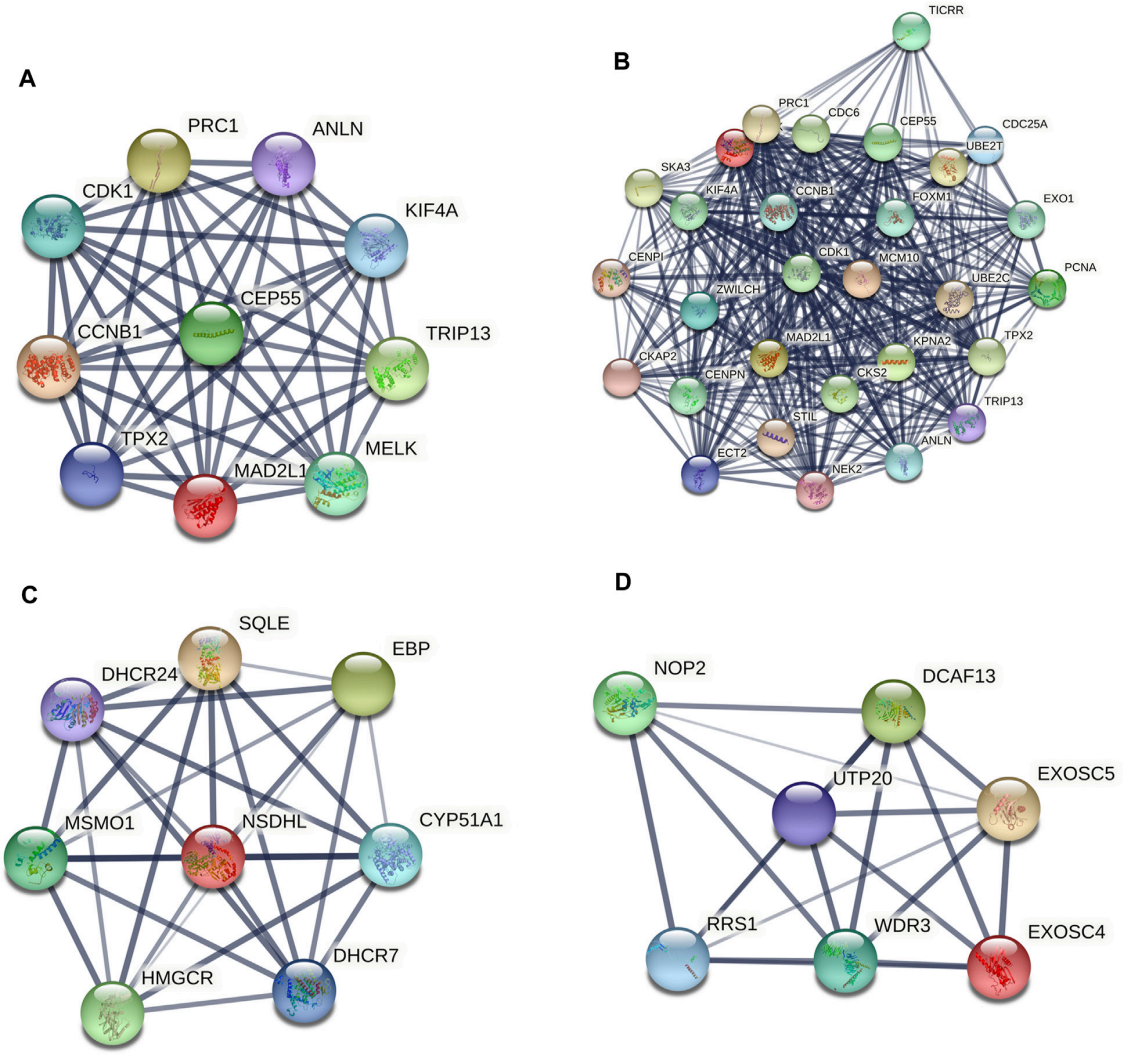
The protein–protein interaction (PPI) network of the top 10 hub genes **(A)** and top three modules from the protein-protein interaction network: module 1 **(B)**, module 2 **(C)**, module 3 **(D)**.

A PPI network of the top 10 hub genes were constructed by STRING database (Figure 1 and Table 1). The top 10 hub genes with a high degree of connectivity were as follows: CDK1, CCNB1, MAD2L1, CEP55, TPX2, MELK, TRIP13, KIF4A, PRC1 and ANLN. Based on the GO function and KEGG pathway analysis, we found that CEP55, ANLN, CDK1, CCNB1 and MAD2L1 were enriched in the cell cycle and cell division. To further detect the most important module in this PPI network, the MCODE plug-in was used and the top three modules were selected (Figure 2). KEGG pathway analysis revealed that the top three modules were mainly associated with the cell cycle, mismatch repair, p53 signaling pathway (Table 4).

**TABLE 4:** The enriched pathways of top three modules.

	Term	Count	%	*p*Value	Genes	FDR
Module 1	hsa04110:Cell cycle	6	1.55	1.67E-07	CCNB1, CDK1, CDC6, MAD2L1, PCNA, CDC25A	0.001
	hsa04914:Progesterone-mediated oocyte maturation	4	1.04	0.01	CCNB1, CDK1, MAD2L1, CDC25A	0.09
	hsa04114:Oocyte meiosis	3	0.78	0.01	CCNB1, CDK1, MAD2L1	5.32
	hsa03430:Mismatch repair	2	0.52	0.03	EXO1, PCNA	17.36
	hsa04115:p53 signaling pathway	2	0.52	0.09	CCNB1, CDK1	43.23
Module 2	bta00100:Steroid biosynthesis	6	7.32	1.51E-12	EBP, CYP51A1, SQLE, DHCR7, DHCR24, NSDHL	3.42E-10
Module 3	cfa03018:RNA degradation	2	2.50	0.01	EXOSC4, EXOSC5	1.18

### The Expression and Survival Analysis of the 10 Hub Genes

**FIGURE 3:**
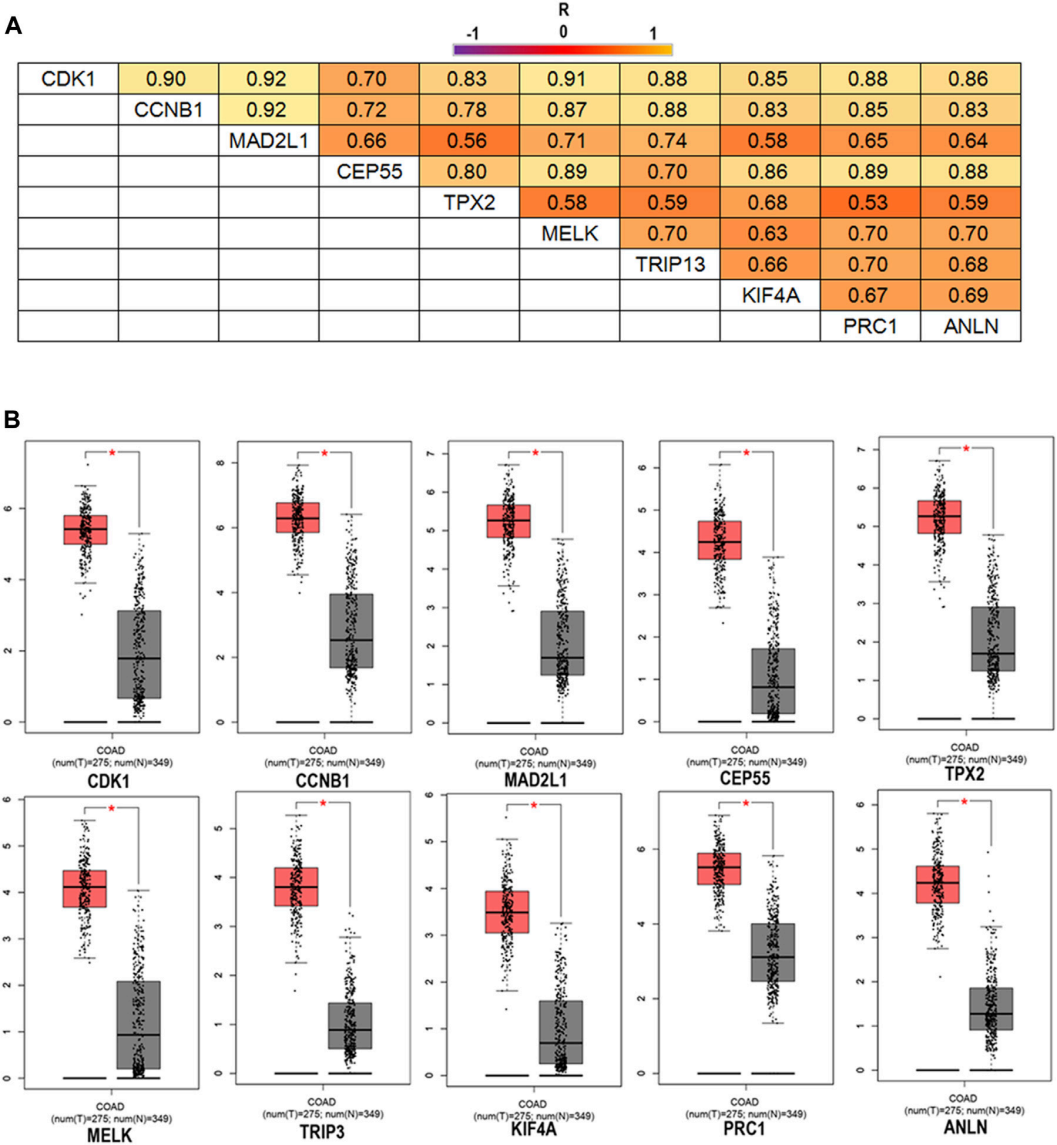
The correlation analysis of the 10 hub genes in CRC **(A)** and expression levels of the 10 hub genes in CRC compared to the normal samples **(B)**. Notes: R is the Pearson correlation coefficient. Abbreviations: CRC, colorectal cancer.

**FIGURE 4:**
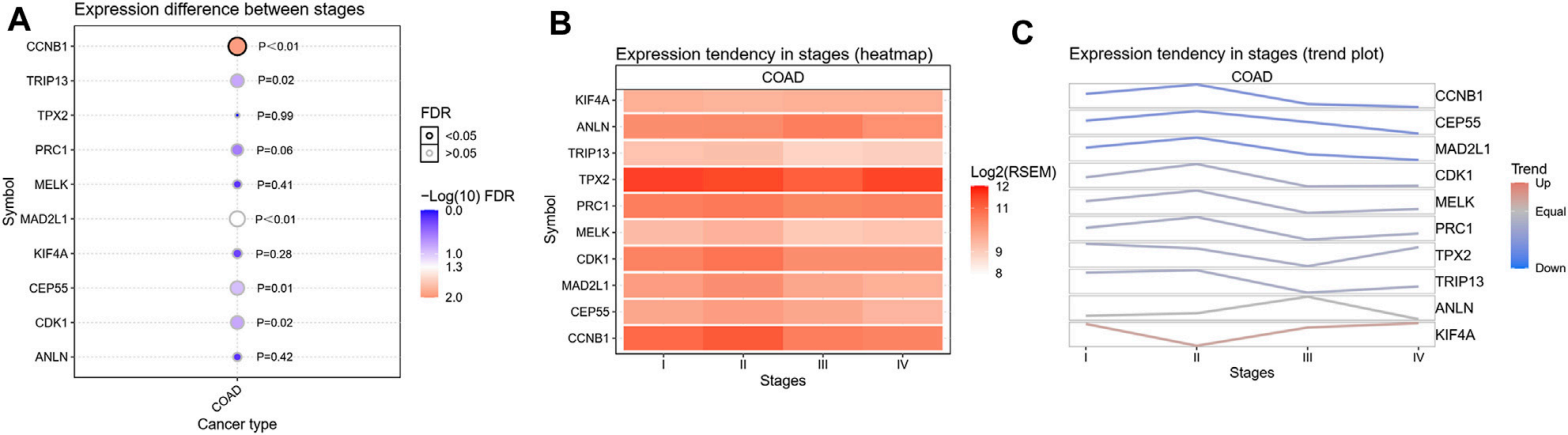
The correlation analysis of the 10 hub genes’ expression and CRC clinic stages. **(A)** Expression difference between stages, **(B)** Expression tendency in stages (heatmap), **(C)** Expression tendency in stages (trend plot).

**FIGURE 5:**
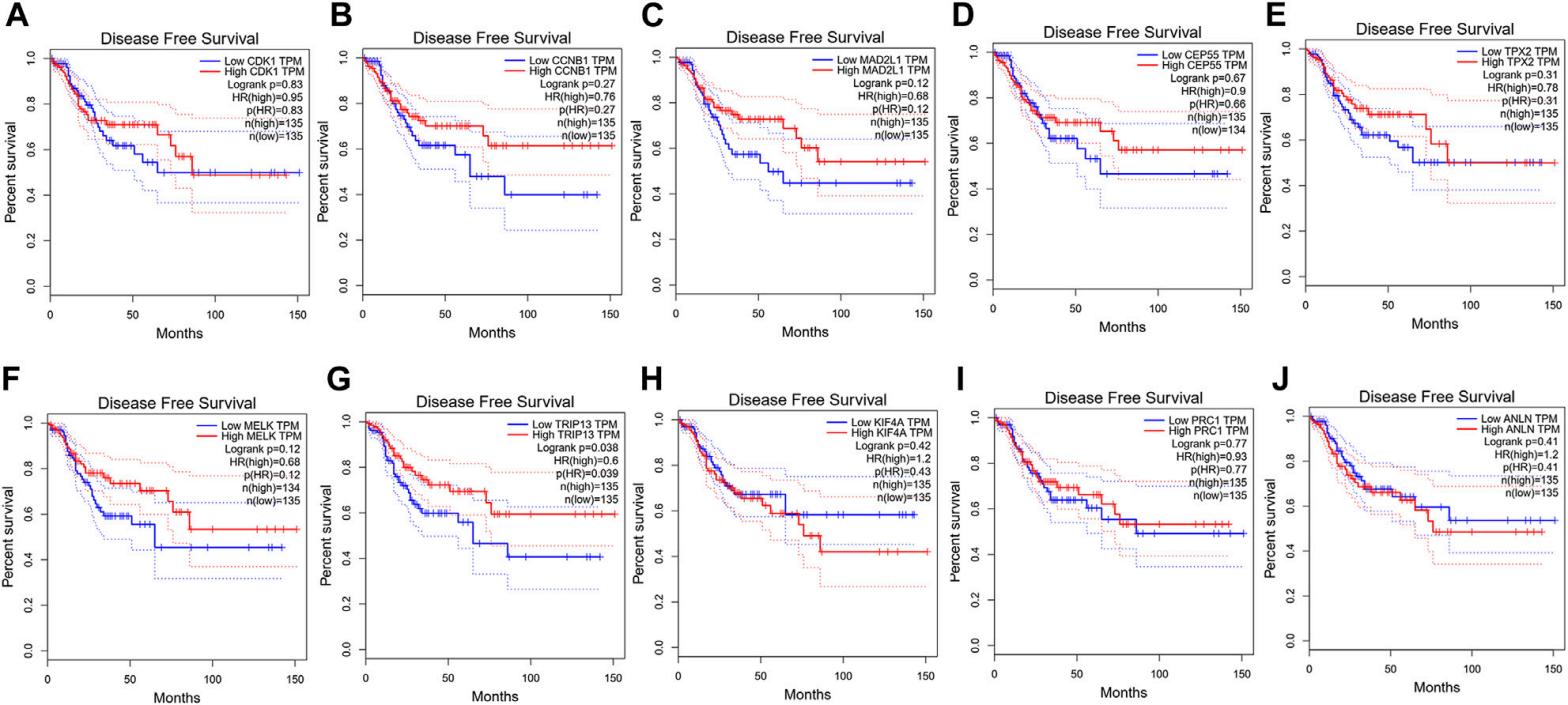
Disease-free survival analysis of CRC in relation to the expression of the 10 hub genes. **(A)** CDK1, **(B)** CCNB1, **(C)** MAD2L1, **(D)** CEP55, **(E)** TPX2, **(F)** MELK, **(G)** TRIP13, **(H)** KIF4A, **(I)** PRC1 and **(J)** ANLN.

To confirm the reliability of the 10 identified hub genes from the datasets, GEPIA was used to verify the correlation between them. We found that these 10 hub genes were obviously positively correlated with each other in CRC (Figure 3A). GEPIA was also used to determine the expression levels of the top 10 hub genes in CRC. Figure 3B showed that these genes were all significantly overexpressed in the colon cancer (COAD) samples compared to the normal samples. GSCA was used to analyze the correlation of the 10 hub genes’ expression and CRC clinic stages. Figures 4A–C showed that the expression of CEP55, CCNB1, CDK1 and TRIP13 was significantly different between different CRC stages (*p* ＜ 0.05). Furthermore, the disease-free survival analysis of the 10 genes was obtained from GEPIA. Among these hub genes, only high expression of TRIP13 was significantly associated with a favorable outcome of CRC (HR: 0.60, *p* = 0.04) (Figure 5). Since these hub genes were validated in the CRC samples from TCGA, further verification of these hub genes in Fn-infected CRC was needed.

### Validation Based on Fn-Infected and Non-Infected Caco-2 Cells

**FIGURE 6:**
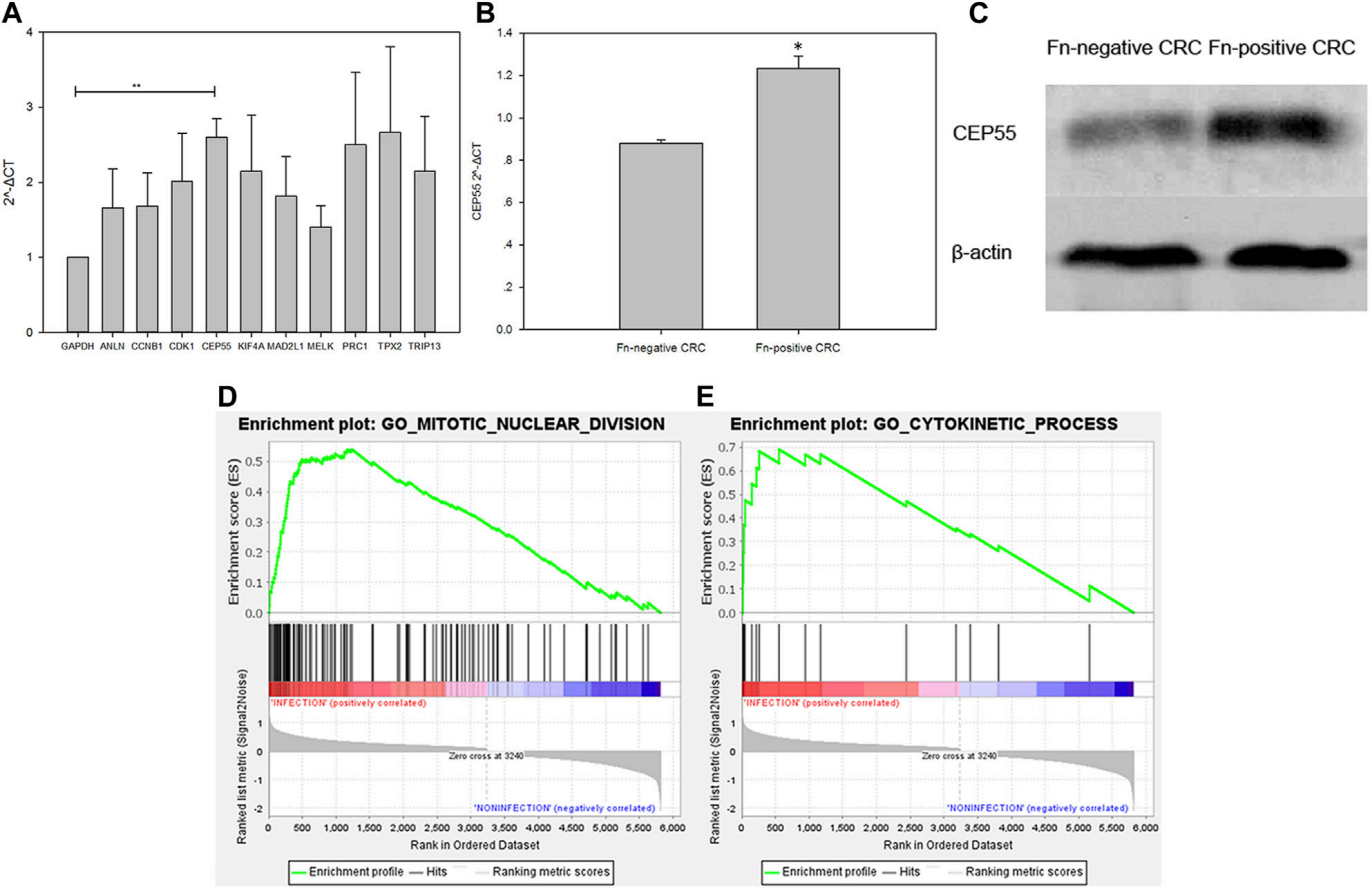
Validation of the 10 upregulated hub genes in Fn-infected Caco-2 and CEP55 in Fn-infected CRC **(A**,**B**,**C)** and GSEA exhibition of two representative functional gene sets enriched in CRC with CEP55 highly expressed **(D**,**E)**.

**FIGURE 7:**
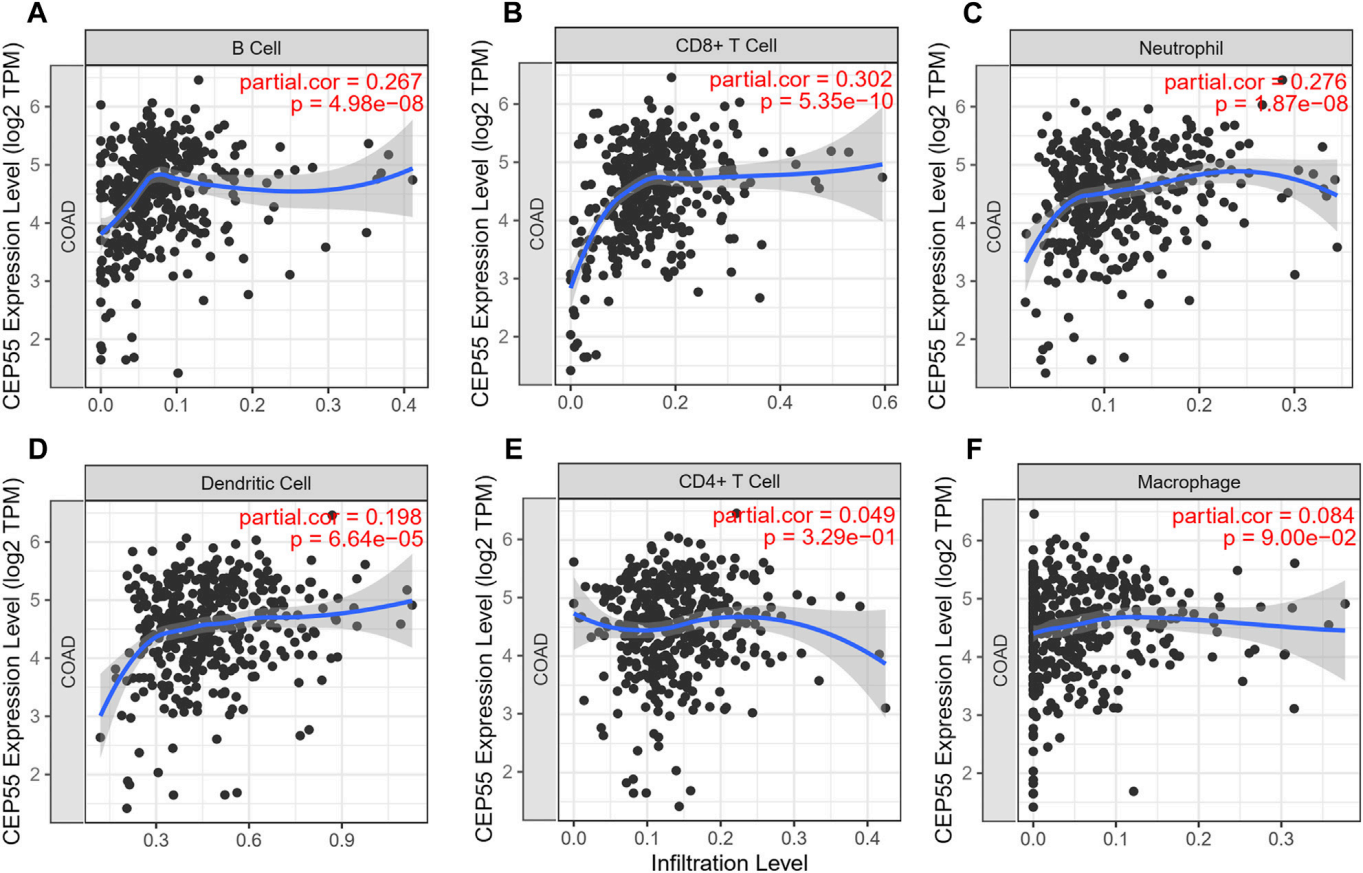
The correlations of CEP55 mRNA expression with immune cell infiltration in CRC. **(A)** B cells, **(B)** CD8 + T cells, **(C)** neutrophils, **(D)** dendritic cells, **(E)** CD4 + T cells and **(F)** macrophages.

The expression level of the 10 identified hub genes was further validated in Fn-infected Caco-2 cells cultured by our group. We found that, as shown in Figure 6A, compared with the Fn-non-infected Caco-2 cells, the relative expression levels of 10 hub genes in Fn-infected Caco-2 cells were increased. However, only the relative expression of CEP55 was significantly increased (*p* = 0.008). The relative mRNA and protein expression of CEP55 in Fn-infected and non-infected CRC specimens was also compared. As shown in Figures 6B,C, the relative expression of CEP55 in Fn-infected CRC group was significantly higher than that in Fn-non-infected CRC group (*p* = 0.023). The expression of CEP55 is similar in Fn-infected Caco-2 cells and Fn-infected CRC, which suggested that our results for this gene expression are reliable. To gain further insight into the functions of the CEP55, GSEA was conducted to map CEP55 into the GO database. The top two pathways were “mitotic nuclear division” and “cytokinetic process” (Figures 6D,E). The TIMER database was also searched to estimate the correlations of CEP55 mRNA expression with immune cell infiltration in CRC. As illustrated in Figure 7, the expression of CEP55 was positively correlated with immune infiltration of B cells, CD8^+^ T cells, neutrophils and dendritic cells. Therefore, we speculated that high CEP55 expression might affect Fn-infected colon cancer cells proliferation and differentiation through mitotic nuclear division, cytokinetic process and immune infiltration.

### Knockdown of CEP55 Suppressed Fn-Infected Caco-2cells Growth by Impairing Cell Cycle Progression and Inducing Cell Apoptosis

**FIGURE 8:**
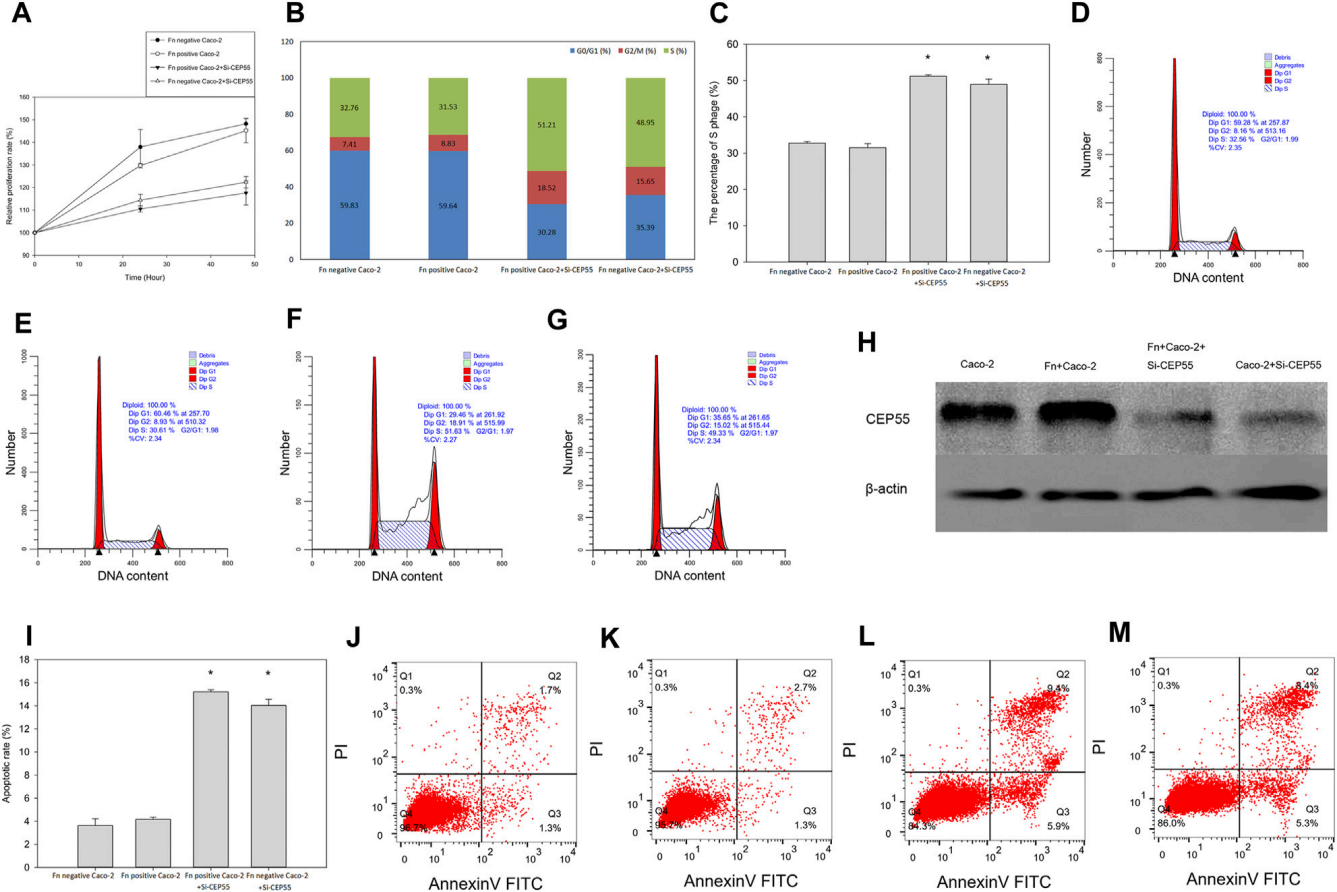
CEP55 knockdown suppressed Fn-infected Caco-2 cells proliferation by impairing cell cycle progression and inducing apoptosis. **(A-H)**, Cell proliferation analysis and the CEP55 protein expression, **(I-M)**, Apoptotic analysis.

To determine whether CEP55 could be a crucial component in Fn induced CRC, CEP55 was inactivated by using siRNAs in Fn-infected Caco-2 cells. We found that, compared to the control group, the CEP55 knockdown significantly inhibited cell proliferation (Figure 8A) and the CEP55 protein expression (Figure 8H). Knockdown of CEP55 resulted in the increase of cells number in S-phase and the decrease of cells population in G1+G2 phase (Figures 8B–F), which indicated that CEP55 knockdown prevented cell passage from the S phase into the G2 phase. Therefore, CEP55 was shown to promote S/G2 phase transition. The apoptotic assay results indicated that the apoptotic cells significantly increased in Fn-infected Caco-2 cells with si-CEP55 transfection (Figures 8I–L). These data indicated that CEP55 knockdown could impair cell cycle progression and induce cell apoptosis in Fn-infected Caco-2 cells. However, we also found that CEP55 knockdown had a similar effect on Fn-non-infected Caco-2 as on Fn-infected Caco-2 cells (Figures 8A–C,G,I,M), which meant that the effect of CEP55 on Fn-infected Caco-2 cells was not specific, other stimuli might also have the effects to upregulate the expression of CEP55.

### The Correlation Between CEP55 Expression and Fn Amount in Fn-Infected CRC Samples

The age, gender, tumor location, tumor size, clinical stage, differentiation grade and distant metastasis of Fn-infected CRC patients are shown in Table 5. The expression of CEP55 and Fn amount in these samples was detected and the correlation between CEP55 and Fn was also analyzed. As shown in Supplementary Figure S1A, Pearson correlation was significant between the expression of CEP55 and Fn amount (R = 0.561; *p* < 0.01). The expression of CEP55 increased along with the increase of Fn amount in Fn-infected CRC.

**TABLE 5:** The baseline characters of Fn-infected CRC patients.

	Patients number	%
Age		
≤60	10	33.3
>60	20	66.7
Gender		
Male	13	43.3
Female	17	56.7
Site		
Left	22	73.3
Right	8	26.7
Tumor size (cm)		
≤4	11	36.7
>4	19	63.3
Stage		
Stages I & II	20	66.7
Stage III & IV	10	33.3
Tumor differentiation		
Moderate+well	23	76.7
Poor	7	23.3
Metastasis		
Yes	14	46.7
No	16	53.3

### The CEP55 Expression and Clinicopathology of Fn-Infected CRC

The relationship between CEP55 expression and clinicopathology of 30 patients with Fn-infected CRC was analyzed (Table 6). The median value of 2^-ΔCT (1.59) was chosen as the cutoff level. The high CEP55 group was defined as those higher than the median value, and the low CEP55 group was defined as those lower than the median value. The proportions of poorly differentiated tumor and distant metastasis were significantly higher in the high CEP55 group (*p* = 0.031, *p* = 0.028), whereas the proportions of old age, male gender, tumor location, tumor size and clinical stage were not significantly different between these two groups. The odds ratio (OR) and cumulative survival rate of high CEP55 expression in Fn-infected CRC patients were also calculated (Table 7). The OR was 12.25 (95%CI: 1.27–118.36) for tumor differentiation, and 5.50 (95%CI: 1.15–26.41) for metastasis in high CEP55 expression. The cumulative survival rate of Fn-infected CRC with high expression of CEP55 was significantly decreased (*p* = 0.038), as shown in Supplementary Figure S1B. These results suggested that Fn infection might promote the progression and metastasis of CRC through overexpression of CEP55.

**TABLE 6:** The clinical features of low and high CEP55 expression in Fn-infected CRC patients.

	Patients number		*p* value
	Low CEP55	High CEP55	
Age			
≤60	6	4	0.44
>60	9	11	
Gender			
Male	6	7	0.71
Female	9	8	
Site			
Left	12	10	0.41
Right	3	5	
Tumor size (cm)			
≦4	5	6	0.71
＞4	10	9	
Stage			
Stages I & II	12	8	0.12
Stage III & IV	3	7	
Tumor differentiation			
Moderate+well	14	9	0.03
Poor	1	6	
Metastasis			
Yes	4	10	0.03
No	11	5	

**TABLE 7:** The Odds ratio of high/low CEP55 expression in Fn-infected CRC patients.

	High CEP55 vs. low CEP55	95%CI	*p* value
	OR		
Age			
≤60/>60	0.58	0.14–2.48	0.47
Gender			
Female/Male	1.83	0.39–8.57	0.44
Stage			
Stages I & II/III & IV	3.50	0.69–17.71	0.13
Site			
Left/Right	2.00	0.38–10.51	0.41
Tumor differentiation			
Moderate+Well/Poor	12.25	1.27–118.36	0.03
Metastasis			
No/Yes	5.50	1.15–26.41	0.03
Tumor size (cm)			
≤4/＞4	1.33	0.30–5.91	0.71

## Discussion

It has been increasingly accepted that CRC is the most relevant cancer type associated with Fn infection (Shang and Liu, 2018). To date, several studies have reported the promoting effects of Fn on CRC initiation and progression (Rubinstein et al., 2013; Flanagan et al., 2014; Park et al., 2016; Chen et al., 2017; Yang et al., 2017; Yamaoka et al., 2018). However, the mechanism of Fn infection in CRC is not clearly and fully understood. In the present study, we mined microarray data obtained from a cellular model of Caco-2 cells that were infected by Fn from the GSE102573 dataset of the GEO database. We identified 10 hub genes potentially involved in Fn induced tumor initiation and progression. Our results further suggested that CEP55 might play an important role in Fn-infected colon cancer cell growth and cell cycle progression.

A total of 450 DEGs were identified, including 272 upregulated genes and 178 downregulated genes. To better explore these DEGs, we carried out GO function and KEGG pathway analysis of these DEGs. GO analysis showed that the upregulated DEGs were particularly enriched in “cell cycle phase,” “cell cycle process,” “cell cycle and mitotic cell cycle” and “M phase,” while the downregulated DEGs were involved in “cell adhesion” and “biological adhesion.” In addition, the KEGG pathways for the upregulated DEGs included the cell cycle and one carbon pool by folate, while the pathways of the downregulated DEGs were enriched in chemokine signaling pathway and metabolism of xenobiotics by cytochrome P450. PPI network module analysis could provide a visible framework for a better understanding of the functional organization of the proteome (Liu et al., 2009). The enriched pathways of the top three modules showed that Fn-infected Caco-2 cells were mainly associated with the cell cycle, mismatch repair and p53 signaling pathway, which are the major pathways involved in the carcinogenesis of CRC.

10 DEGs with high connectivity were selected as hub genes for PPI network analysis. These hub genes were all belong to upregulated DEGs. By analyzing the correlations and expression levels in GEPIA, we found that these hub genes were obviously positively correlated and significantly overexpressed in CRC samples. GSCA analysis found that the expressions of CEP55, CCNB1, CDK1 and TRIP13 were significantly increased in stage II of CRC, therefore, these genes, especially CEP55, may be related to the development and proliferation of early CRC. Further analysis using GEPIA exhibited that only TRIP13 was significantly associated with CRC survival, the reason for this might be that different inclusion criteria for high and low mRNA expression, clinical stages and pathological grading are applied in the prognosis analysis. We further searched the literature in PubMed for associations among the 10 hub genes in CRC. Recent studies revealed that upregulated CDK1 promotes CRC cell proliferation via the inhibition of the p53 pathway (Gan et al., 2017), CCNB1 overexpression exerts oncogenic role in CRC cells by phosphorylating CDK1 (Fang et al., 2014), high expression of MAD2L1 drives aneuploidy and carcinogenesis in CRC (Ding et al., 2020), TPX2 promotes proliferation and tumorigenicity of colon cancer cells (Wei et al., 2013), MELK overexpression is significantly correlated with advanced tumor stage and further lymph node metastasis (Gong et al., 2018), TRIP13 and KIF4Acould promote CRC cell proliferation, invasion and migration and subcutaneous tumor formation (Hou et al., 2018; Sheng et al., 2018), overexpression of PRC1 and ANLN facilitate CRC tumor growth and proliferation (Wang et al., 2016; Xu et al., 2020). The increase expression of these hub genes is closely related to the occurrence and development of CRC.

Recent studies have confirmed that Fn could significantly downregulate the expression of CDK1 in gingival keratinocytes (Bhattacharya et al., 2014). *Aggregatibacter actinomycetemcomitans*, *Porphyromonas gingivalis* and *Neisseria meningitides* infections were found to down-regulate CCNB1 and MAD2L1 expression in gingival epithelial cells and brain endothelial cells (Oosthuysen et al., 2016; Zhu et al., 2018). However, we did not find any evidence for a significant correlation between TPX2, MELK, TRIP13, KIF4A, PRC1, ANLN and bacterial infection. Further studies are needed to verify the relationship between these genes and bacterial infection.

We speculated that Fn infection could dysregulate the above-mentioned hub genes through various signaling pathways, therefore we further conducted qRT-PCR analysis to verify the microarray results. We found that although the expression of these 10 hub genes was all higher than the control, only CEP55 was significantly increased (*p* < 0.05). CEP55, also known as c10orf3 or FLJ10540, was initially identified as a major player in abscission of cytokinesis. Bioinformatics analysis found that the top two pathways of CEP55 involved in CRC were “mitotic nuclear division” and “cytokinetic process,” and the expression of CEP55 was positively correlated with immune infiltration of B cells, CD8^+^ T cells, neutrophils and dendritic cells which play an important role in the chronic Fn infection. Therefore, we speculated that high CEP55 expression might affect Fn-infected colon cancer cells proliferation and differentiation through mitotic nuclear division, cytokinetic process and immune infiltration. Recently studies have demonstrated that CEP55 could promote cancer cell stemness and tumor formation through regulating the PI3K/AKT pathway. Clinically, Cep55 has also been found to be overexpressed in many cancer types, and its overexpression has been strikingly associated with tumor stage and metastasis (Tandon and Banerjee, 2020). We demonstrated that, compared with Fn-non-infected Caco-2 cells, the relative expression of CEP55 was significantly higher in Fn-infected Caco-2 cells and knockdown of CEP55 inhibited cell proliferation and induced cell apoptosis in these cells. Correlation analysis exhibited that the expression of CEP55 was positively correlated with the Fn amount in Fn-infected CRC patients, and these patients with high CEP55expression had an obviously poorer differentiation, worse metastasis and decreased cumulative survival rate. These results suggested that Fn-infection might cause progression and metastasis of CRC through overexpression of CEP55 and CEP55 has the potential to be a new biomarker for diagnosis and prognosis of Fn-infected CRC.

It has been reported that the expression of CEP55 in peripheral blood cells is significantly up-regulated in septicemia and abdominal infection that caused by bacterial infection (Alonso et al., 2017; Lu et al., 2020), which means that bacterial infection could increase the expression of CEP55. Recent studies have also found that Fn can cause DNA damage and promote cell proliferation by downregulating the expression of Ku70/p53, whereas the expression of CEP55 could be up-regulated through down-regulation of p53 (Chang et al., 2012; Geng et al., 2019). Overexpression of CEP55 was found to promote proliferation, metastasis and invasion of esophageal squamous cell carcinoma by activating PI3K/Akt signaling pathway (Jia et al., 2018). Therefore, we infer that Fn infection might upregulate the expression of CEP55 through downregulating p53, and the upregulation of CEP55 might lead to excessive proliferation, invasion and metastasis of CRC through activating PI3K/Akt signaling pathways. We will further verify the expression of CEP55 in Fn-infected CRC cell lines, animal models and patients and elucidate the molecular mechanism of CEP55 in the proliferation, invasion and metastasis of tumor cells induced by Fn infection.

We acknowledge some limitations of our present study. In this study, DEGs in response to Fn infection obtained from bioinformatics analysis were shown and candidate genes associated with tumorigenic properties were analyzed. And we primarily verified the expression of CEP55 in Fn-infected CRC patients, therefore, more functional assays should be applied to explore and validate the functional roles of CEP55 in Fn-infected CRC. In addition, though we have validated the expression of these hub genes in a small clinical dataset of Fn-infected CRC, other datasets derived from larger scale clinical samples which contain different intestinal conditions and Fn infection prevalence rates should be applied for further validation and evaluation.

In summary, using multiple bioinformatics analyses and qRT-PCR validation, our present work identified 10 hub genes as DEGs. These upregulated DEGs are significantly enriched in several pathways that are mainly associated with the cell cycle and mitotic cell cycle in Fn-infected CRC, and might play critical roles in the development and progression of Fn induced CRC. High expression of CEP55 has been demonstrated to be involved in Fn-infected colon cancer cell growth and cell cycle progression, and could be used as a new diagnostic and prognostic biomarker for Fn-infected CRC.

## Conclusion

In this study, using multiple bioinformatics analyses, we identified 10 hub genes that were significantly enriched in the cell cycle, mismatch repair and p53 signaling pathway in Fn-infected Caco-2 cells. Moreover, the expression level of CEP55 was significantly increased in Fn-infected CRC, and knockdown of CEP55 suppressed Fn-infected colon cancer cell growth by impairing cell cycle and apoptosis progression. Our findings suggest that CEP55 plays an important role in Fn-infected colon cancer cell growth and cell cycle progression and could be used as a new diagnostic and prognostic biomarker for Fn-infected CRC.

## Data Availability

The datasets presented in this study can be found in online repositories. The names of the repository/repositories and accession number(s) can be found in the article/Supplementary Material.
