# Supplementary figures and images for: Identification and Validation of Potential Candidate Genes of Colorectal Cancer in Response to Fusobacterium nucleatum Infection
Source: Front Genet. 2021 Sep 28;12:690990. doi: 10.3389/fgene.2021.690990 (PMC8508782; doi:10.3389/fgene.2021.690990)

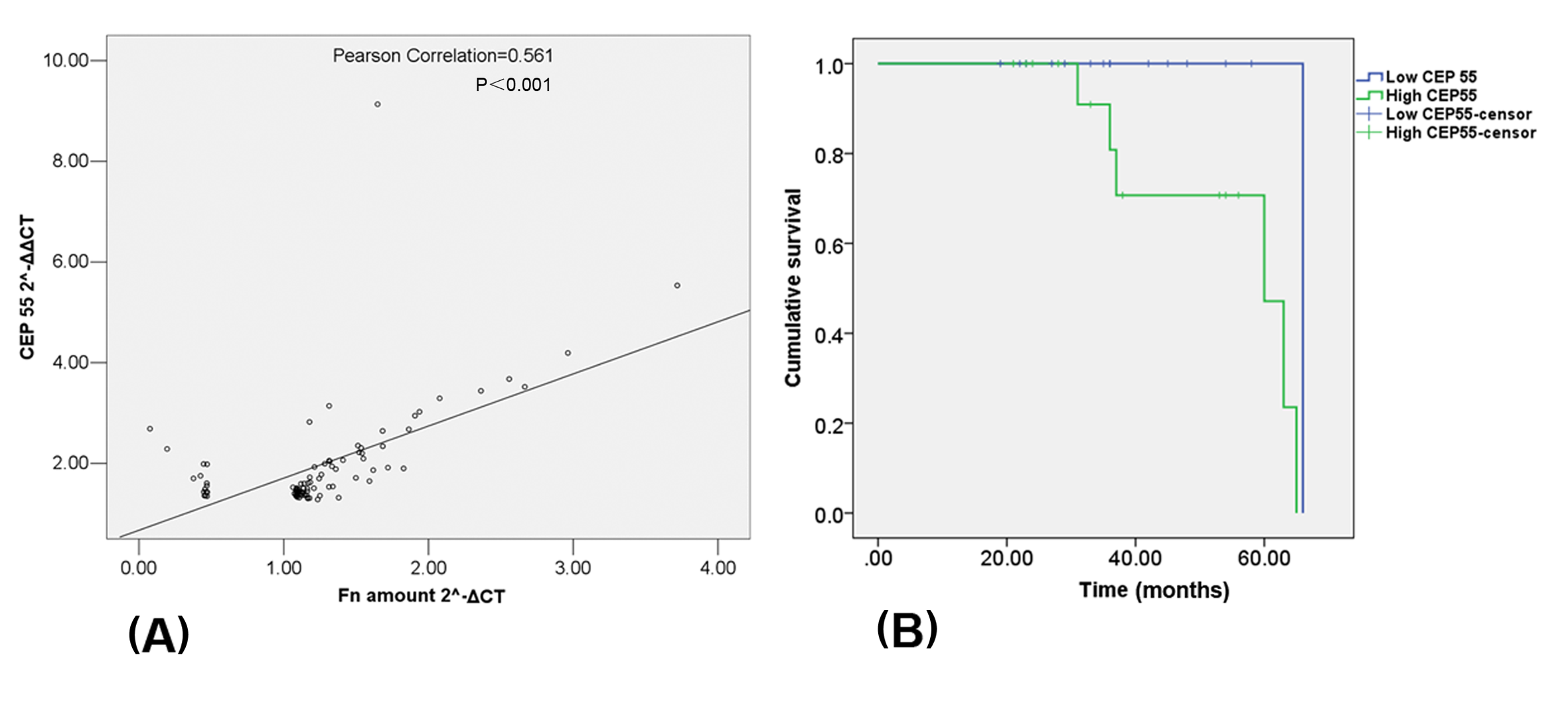

Supplement: Supplementary file 1 [file Image1.TIF]
